# Supplementary material for: Australian Veterinarians’ Perceptions Regarding the Zoonotic Potential of Mycobacterium avium Subspecies Paratuberculosis
Source: Vet Sci. 2020 Mar 19;7(1):33. doi: 10.3390/vetsci7010033 (PMC7157240; doi:10.3390/vetsci7010033)
Supplement: Supplementary file 1 [file vetsci-07-00033-s001.pdf]

veterinary sciences

MDPI

Article

Australian Veterinarians' Perceptions Regarding the Zoonotic Potential of *Mycobacterium avium* Subspecies *Paratuberculosis*

Kamal R. Acharya, Karren M. Plain, Richard J. Whittington and Navneet K. Dhand *

Sydney School of Veterinary Science, Faculty of Science, The University of Sydney, 425 Werombi Road, Camden, 2570, New South Wales, Australia; kach8080@uni.sydney.edu.au (K.R.A.); karren.plain@sydney.edu.au (K.M.P.); richard.whittington@sydney.edu.au (R.J.W.)

* Correspondence: navneet.dhand@sydney.edu.au; Tel: +61-2-9351-1669; Fax: +61-2-9351-1618

Received: 17 February 2020; Accepted: 9 March 2020; Published: date

Abstract: Public concerns over exposure to *Mycobacterium avium* subspecies *paratuberculosis* (MAP) or MAP components via foods of animal origin could have negative trade consequences, despite the absence of conclusive scientific evidence of a causal association between *Mycobacterium avium* subspecies *paratuberculosis* (MAP) and Crohn's disease (CD). This study was conducted among Australian veterinarians to understand (a) their perceptions regarding the role of MAP in the causation of CD (an ordinal outcome), and (b) their consideration of the adoption of the precautionary principle against Johne's disease (JD; a binary outcome). Ordinal and binary logistic regression analyses were performed to evaluate the association of explanatory variables with the above outcomes, respectively. Almost one-third of the respondents (32.2%) considered that MAP was likely to be involved in the causation of CD whereas more than two-thirds (69.8%) agreed with the adoption of the precautionary principle against JD. Veterinarians who were concerned about exposure to and/or getting infected with MAP were more likely to consider MAP as a causative agent of CD (odds ratio: 7.63; 95% CI: 1.55, 37.63) and favor the adoption of the precautionary principle against JD (odds ratio: 6.20; 95% CI: 1.90, 20.25). Those perceiving MAP as a causative agent of CD were also more likely to favor the adoption of the precautionary principle against JD (odds ratio: 13.2; 95% CI: 1.26, 138.90). The results suggest that Australian veterinarians, particularly those who consider MAP as a causative agent of CD are concerned about exposure to MAP and favor the adoption of the precautionary principle against JD. These findings can be useful for animal health authorities for designing JD control programs and policies.

veterinary sciences

MDPI

Table S1. Frequency table of categorical explanatory variables used in the analysis in the study conducted in Australia in 2016 to investigate the veterinarians' perceptions regarding the zoonotic potential of *Mycobacterium avium* subspecies *paratuberculosis*.

Variables	Categories	Count (%)	N	No response (%)
Mode of survey administration	ACV conference	35 (34.3)		
	ASV conference	22 (21.6)	102	0 (0.0)
	Others	45 (44.1)		
Year of graduation	Before 1980	18 (17.7)		
	1980 to 2000	47 (46.1)	102	0 (0.0)
	After 2000	37 (36.3)		
Gender	Female	33 (32.7)	101	1(1.0)
	Male	68 (67.3)		
Level of education	Bachelor's degree	57 (55.9)	102	0 (0.0)
	Higher Education	45 (44.1)		
Geography of work	New South Wales	39 (38.2)		
	Victoria	31 (30.4)	102	0 (0.0)
	Others	32 (31.4)		
Location of clients	Rural only	88 (86.3)	102	0 (0.0)
	Others	14 (13.7)		
Type of work	Private practice only	43 (44.3)		
	Private and other practice	46 (47.4)	97	5 (5.0)
	No private practice	8 (8.3)		
Animal worked with	Food animals	58 (63.1)		
	Non-food animals	20 (21.7)	92	10 (9.9)
	Both	14 (15.2)		
Laboratory used	Private	51 (53.1)	96	6 (5.9)
	Others	45 (46.9)		
Continuing education on JD	Yes	58 (57.4)	101	1 (1.0)
	No	43 (42.6)		
Market assurance program training	Yes	63 (62.4)	101	1 (1.0)
	No	38 (37.6)		
Consultation on JD control and management on farm	Yes	25 (29.1)		
	Sometimes	46 (53.5)	86	16 (15.7)
	No	15 (17.5)		
Consultation on JD diagnosis on farm	Yes	15 (17.3)		
	Sometimes	53 (61.0)	87	15 (14.8)
	No	19 (21.8)		
Diagnosis of JD[a]	Single species	29 (33.3)		
	Multi-species	38 (43.7)	87	15 (14.8)
	None	20 (23.0)		
Awareness on BJD management programs in Australia	Agree	41 (51.3)		
	Neutral	20 (25.0)	80	22 (21.6)
	Disagree	19 (23.8)		
	Yes	64 (78.1)	82	20 (19.7)

veterinary sciences

MDPI

Variables	Categories	Count (%)	N	No response (%)
Mode of survey administration	ACV conference<br>ASV conference<br>Others	35 (34.3)<br>22 (21.6)<br>45 (44.1)	102	0 (0.0)
Awareness on BJD review by Animal Health Australia	No	18 (22.0)		
Awareness of OJD regulatory provisions	Agree<br>Neutral<br>Disagree	36 (46.8)<br>17 (22.1)<br>24 (31.2)	77	25 (24.6)
Concern index on MAP exposure and infection	Concerned/highly concerned<br>No/some concerns	61 (71.8)<br>24 (28.2)	85	17 (16.7)
Know a Crohn's disease patient	Yes<br>No	59 (67.8)<br>28 (32.2)	87	15 (14.8)
Updated knowledge on JD	Agree<br>Neutral<br>Disagree	41 (47.1)<br>25 (28.7)<br>21 (24.1)	87	15 (14.8)
Agreement Index on MAP as a zoonotic agent	Agree<br>Neutral<br>Disagree	34 (39.5)<br>10 (11.6)<br>42 (48.8)	86	16 (15.7)
Proportion of clients with JD infected properties	High<br>Medium<br>Low	25 (33.8)<br>24 (32.4)<br>25 (33.8)	74	28 (27.5)
Proportions of clients who had initiated control programs or were intending to control JD	High<br>Medium<br>Low	22 (30.6)<br>25 (34.7)<br>25 (34.7)	72	37 (36.3)

[a] Diagnosis made in single species (bovine or ovine), multi-species (both bovine and ovine) and none; ACV: Australian Cattle Veterinarians; ASV: Australian Sheep Veterinarians; JD: Johne's disease; OJD: Ovine Johne's disease; BJD: Bovine Johne's disease MAP: *Mycobacterium avium* subspecies *paratuberculosis*.
